# Artificial Intelligence and Human Trust in Healthcare: Focus on Clinicians

**DOI:** 10.2196/15154

**Published:** 2020-06-19

**Authors:** Onur Asan, Alparslan Emrah Bayrak, Avishek Choudhury

**Affiliations:** 1 School of Systems and Enterprises Stevens Institute of Technology Hoboken, NJ United States

**Keywords:** human-AI collaboration, trust, technology adoption, FDA policy, bias, health care

## Abstract

Artificial intelligence (AI) can transform health care practices with its increasing ability to translate the uncertainty and complexity in data into actionable—though imperfect—clinical decisions or suggestions. In the evolving relationship between humans and AI, trust is the one mechanism that shapes clinicians’ use and adoption of AI. Trust is a psychological mechanism to deal with the uncertainty between what is known and unknown. Several research studies have highlighted the need for improving AI-based systems and enhancing their capabilities to help clinicians. However, assessing the magnitude and impact of human trust on AI technology demands substantial attention. Will a clinician trust an AI-based system? What are the factors that influence human trust in AI? Can trust in AI be optimized to improve decision-making processes? In this paper, we focus on clinicians as the primary users of AI systems in health care and present factors shaping trust between clinicians and AI. We highlight critical challenges related to trust that should be considered during the development of any AI system for clinical use.

## Introduction

Artificial intelligence (AI), which has been introduced as a technology to improve decision-making involving uncertainty and complexity in systems, has the potential to transform health care practices [1,2]. The role of humans in the practical applications of AI is often overlooked. The development of automated systems to augment human decision-making dates to the 1950s with the Fitts list, which identifies the complementary capabilities of humans and automated systems [3]. The Fitts list includes 11 statements asserting that humans are better at detection, perception, judgment, induction, improvisation, and long-term memory, while automated systems are better at power/speed, computation, replication, simultaneous operation, and short-term memory [4]. Several studies have shown that automated systems may or may not improve human decision-making, depending on whether or how human factors are accounted for in their design [5].

As AI is rapidly developing, unlike other technologies, there is an absence of a clear definition of the process, functioning, and role of AI [6]. Trust is a crucial factor influencing interactions between human beings, including their interactions with AI. Understanding the trust dynamics between AI and humans is crucial, particularly in the field of healthcare, where life is at risk. In this paper, we discuss the impact of trust on the dynamic interactions between AI and clinicians, highlight the key factors that influence trust relationships and identify key challenges and future research directions in the health care domain. While the users of AI systems can be diverse, including patients and insurance providers, the focus of this paper is limited to the domain experts in healthcare, ie, clinicians. We acknowledge that trust relationships could significantly differ for patients and insurance providers.

## Definitions

### What Is AI?

The term AI has been used in many ways in computer science, engineering, and healthcare. Broadly, it can be defined as a computer program that can make intelligent decisions [7]. This definition includes computer programs that operate with predefined rules and data-driven models. This paper distinguishes these two by referring to the former as *automation*, which can be used to make well-defined and repetitive decisions. While automated systems have been used to augment or replace human operation in health care [8], the generalizability of the process and existence of intelligence in automation can be questionable. The focus of this research is the latter, which is a process to make health care decisions using a mathematical model built on prior or real-time data. Existing literature in the machine learning field provides relatively successful methods to train such mathematical models that learn useful knowledge from data [9-11]. In this paper, we refer to *a computer process that algorithmically makes optimal decisions based on multiple criteria using one or more machine learning-based models* as AI. While trust has been studied in the context of automation [12], the deterministic (ie, consistently providing the same output for a particular input) and relatively predictable nature of automation is an important distinction from our definition of AI, which has implications for trust research.

### What Is Trust?

Interpersonal trust is a human belief (or referred to as an attitude in some sources [12]) that is broadly defined based on three main dimensions, namely, benevolence, integrity, and ability [13]. This attitude may be intrinsically formed based on the user’s own experience with the system of interest or may stem from an extrinsic source such as the reputation of the system in the user’s social circle [14]. Studies highlight some differences between interpersonal trust and trust in technical systems because technical systems may lack intentionality, which is relevant to honesty and benevolence [12]. A user’s perception of an AI system’s ability remains a significant dimension for trust in AI systems, which depends on the quality of the input data, the mathematical problem representation, and the algorithms used in the decision-making. The level of trust in AI has a significant impact on how much users rely on AI [12], and hence the efficacy of health care decisions. However, the level of trust in AI may not necessarily have a positive correlation with clinical or patient outcomes.

## AI in Health Care

AI has shown significant potential in the area of mining medical records, designing treatment plans [15], robotics mediated surgeries [16], medical management and supporting hospital operations, clinical data interpretation [17], clinical trial participation [18,19], image-based diagnosis [20], preliminary diagnosis [21], virtual nursing [22], and connected health care devices [23]. In addition to these applications, significant investments in AI research [24], as well as recent efforts on regulating the use of AI in the medical domain [24], suggest that AI could become an essential technology to assist decision-making in the medical domain in the foreseeable future.

AI research in health care poses unique challenges compared to other technical domains. Physical system models mathematically describe the underlying technical behavior in engineering applications. However, the lack of such quantitative models in many health care applications such as medical diagnostics (eg, the precise relationships between diseases and their causes) creates a significant challenge. The responses from clinicians for the same clinical cases vary significantly. Therefore, it would be a challenge to train AI-based tools on the subjective responses that carry over individual biases from clinicians without any knowledge of the ground truth. Also, AI research must account for the distinct medical problem characteristics across different health care applications. It may not be possible to generalize a process to train a mathematical model for an AI tailored to the needs of cancer applications to cardiovascular applications, for instance. Further, vulnerable groups such as minorities and patients with disabilities may not be sufficiently represented in the data, and their needs may not be adequately accounted for if these groups are not carefully considered during the design of the AI system. A customized AI process might be necessary for each application depending on the type and amount of data available, the target patient population, the amount of variability and useful information in the data, and the nature of the health care decisions to be made.

Further, health care is a highly regulated space where developing an adaptive AI technology to meet regulatory requirements is an additional challenge. The US Food and Drug Administration (FDA) categorizes Software into three classes: (a) Software as a Medical Device (SaMD), (b) software in a medical device, and (c) software used in the manufacture or maintenance of a medical device. FDA defines SaMD as “… AI/ML-based Software, when intended to treat, diagnose, cure, mitigate, or prevent disease or other conditions, are medical devices under the FD&C Act and called Software as a Medical Device” [25]. SaMD ranges from smartphone applications to view radiologic images for diagnostic purposes to Computer-Aided Detection software to post-processing of images to detect breast cancer [26]. FDA has approved several AI-based SaMDs with “locked” algorithms that generate the same result each time for the same input; these algorithms are adaptable but require a manual process for the updates [25,27].

Unlike the standard SaMD model, an adaptive algorithm changes its behavior using a definitive learning process without requiring any manual input. An adaptive algorithm might generate different outputs each time a given set of inputs is received due to learning and updating. A credible validation and verification plan that ensures safe and reliable operation under adaptive behaviors must be a part of the AI design.

AI in health care has two potential advantages to human performance. First, AI can learn from big data (such as incommunicable silos of unstructured information stored in an electronic health record) more efficiently than clinicians. A successful AI system can efficiently extract relevant information from offline or real-time data to assist in improving organizational performance and help clinicians in making informed decisions in real time. Second, AI systems can perform predefined tasks with higher precision. AI can be in a continuous active state without compromising its performance—it does not suffer from burnout as humans do. This feature of AI technology has the potential to revolutionize complicated surgeries. The Da Vinci robotic surgical system can mimic a surgeon’s hand movements with greater precision [28]. Further, existing applications of AI in various domains such as AlphaStar (an AI bot that outperforms an expert player in a video game) and LYNA (an AI capable of detecting breast cancer using images from lymph node biopsies) report successful outcomes comparable to human decision-making [29,30].

There are limitations of AI that might restrict its application in life-critical areas such as healthcare. AI methods require data inputs to be in a structured form, which limits the type of information that can be provided for medical decisions. Even the deep learning methods, which can find a suitable mathematical representation from a given dataset automatically, are trained for a given input type (eg, medical image data) and, once developed, they cannot accept another input type (eg, statements from patients). Also, AI methods generally lack “common sense,” making them unable to identify simple mistakes in data or decisions that would otherwise be obvious to a human being [31]. Therefore, there is significant potential and need for improvement by combining the intuitive and analytical thinking of medical experts [32] with the computational power of AI in a proper human-AI collaboration architecture.

## Trust in Human-AI Collaboration

**Figure 1:**
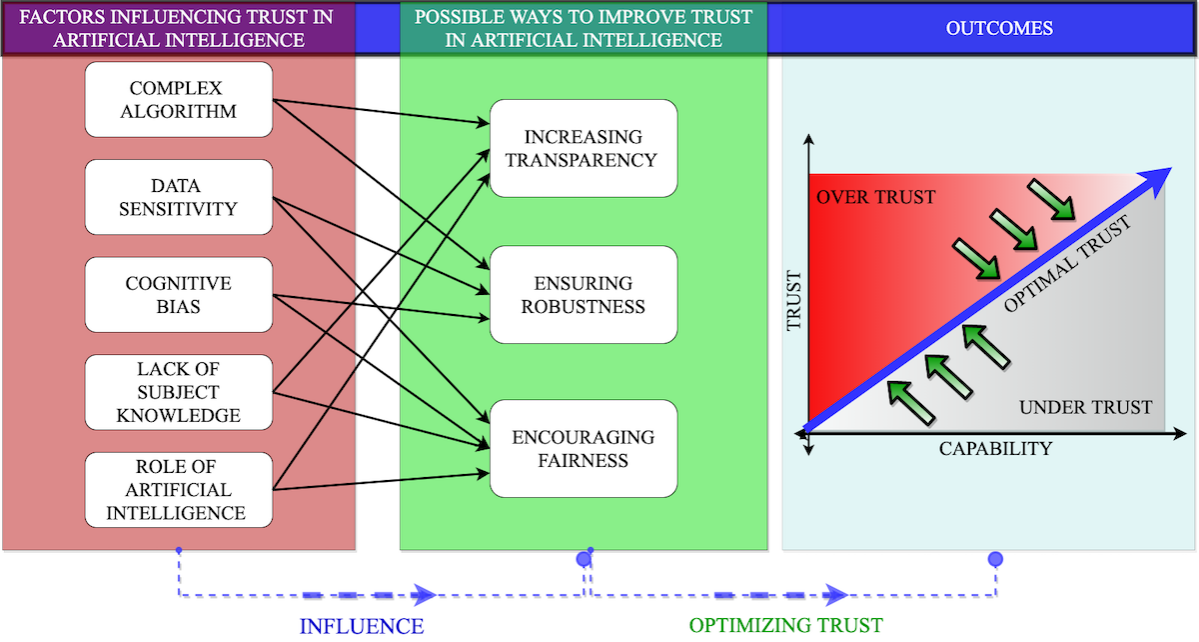
Human factors and trust in artificial intelligence.

Advances in the capabilities of AI will expand the role of this technology from the automation of repetitive and well-defined tasks to guiding decision-making under uncertainty that is currently done exclusively by medical professionals. As health care providers rely more on AI, a proper trust relationship, also referred to as *calibrated* trust [33], becomes a requirement for effective decisions. The deterministic and relatively predictable nature of a typical rule-based software system is an essential factor contributing to the development of user trust. The resulting behavior of a deterministic system can entirely be determined by its initial state and inputs. However, the nondeterministic nature of AI, where an algorithm can exhibit different behaviors in different iterations for the same input, might introduce unique dimensions to the concept of trust. Figure 1 presents our overview of some important factors influencing trust in AI for health care, possible ways to improve trust relationships, and their impact on trust. Note that the purpose of the figure is not to provide an exhaustive list but rather to highlight important issues relevant to trust in AI for health care applications.

Currently, a lack of trust in the AI systems is a significant drawback in the adoption of this technology in healthcare. Trust in AI can be influenced by several human factors such as user education, past experiences, user biases, and perception towards automation, as well as properties of the AI system, including controllability, transparency, and complexity of the model, associated risks, and many others. Among these factors, reliability, which refers to whether the AI technology can perform a task predictably and consistently [34], might be particularly concerning in health care due to the changes in the reliability of AI in the presence of new data [35]. The reliability of an AI technology is conditioned on the user and input data. Considering that an AI system might be trained with insufficient and subjective data from multiple sources, AI could generate biased or overfitted outcomes of which the clinical user might not be aware. These concerns hinder the performance of this technology [36], thus deterring the user’s trust and acceptance of AI systems.

It is also important to note that *maximizing the user’s trust* does not necessarily yield the best decisions from a human-AI collaboration. When trust is at maximum, the user accepts or believes all the recommendations and outcomes generated by the AI system. While in some applications, AI can outperform human decision-making by incorporating data from multiple sources [29,30], the limitations above suggest that unnecessarily high trust in AI may have catastrophic consequences, especially in life-critical applications. Therefore, our study supports the concept of *optimal trust* in which both humans and AI each have some level of skepticism regarding the other’s decisions since both are capable of making mistakes. The amount of skepticism necessary for the most accurate clinical decisions depends on the capability of the human user and the AI system. The development of AI must incorporate mechanisms that will establish and maintain a properly balanced, optimal level of trust from and to the user that matches the capability of the AI system [12].

Developing a healthy trust relationship in human-AI collaboration is a challenge due to the uncertain behavior of a knowledge-based evolving AI system. We posit that the following factors should be incorporated into the development of AI to achieve an optimal level of trust: fairness, transparency, and robustness (Figure 1). It is often assumed that algorithmic decision-making might lead to fairer and more robust outcomes than human judgment [37]. However, algorithms intrinsically discriminate and assign a weight to some factors over others. Moreover, the properties of machine learning algorithms bear the risk to reflect and aggravate underlying data bias, which might unfairly affect members of protected groups based on sensitive categories such as gender, race, and sexual orientation. For instance, a study [38] that implemented machine learning to detect skin cancer used less than 5% data from a dark-skinned patient population to train the model, potentially leading to bias against dark-skinned patients [39]. According to the UK International Commissioner’s Office, AI fairness depends on the effects of the data processing on users (care providers and patients), their expectations as to how their data will be used (clinical and personal data) and the level of transparency provided [40,41] (sharing with other care providers and insurance). Fairness in an AI process is concurrent with bias minimization. Bias, a mismatch between the distribution of training data and a preferred fair distribution, can yield unfair outcomes (prediction/classification). In order to establish fairness in AI systems, biases originating from the subjective responses of clinicians should be identified and curtailed during validation and verification.

Achieving fairness through awareness or transparency can also improve trust [42,43]. Facilitating secured access to patient data can improve transparency [44] in the data and allow patients to validate their information. In our view, transparency fosters an understanding not only of the working principles of AI algorithms but also the algorithmic biases and biases due to underrepresentation. Depending on data availability and quality, an AI system might perform remarkably well at some tasks while performing poorly in others. Revisiting the ‘skin cancer example’ cited above [38], if clinicians are informed about the bias in the training data (eg, underrepresentation of minorities), then it will be easier for them to identify the suitable patient population (eg, Caucasians) on which the AI algorithm can be implemented. Explainable AI is another route to AI transparency that might help clinicians arrive at clinically meaningful explanations about the outcome of AI applications and make informed judgments. However, there are tradeoffs between the explainability and sophistication of an AI algorithm. Explainable AI models such as decision trees intuitively tend to be simple and might not be accurate, especially when dealing with big and complex data. Determining the precise balance between explainability and sophistication is crucial to enhance trust in AI. Finally, robustness is the sensitivity of the decisions made by the AI models to the input dataset. Poor robustness can lead to significant changes in the outcome of an AI model with small perturbations in the input data. Insufficient or erroneous data can impede AI robustness. Proofing AI models against such volatilities can help to build trust in AI systems.

## Future Research Directions

The efficacy of human-AI collaboration is not only a function of the accuracy of the underlying mathematical process behind the AI system but also human factors, including trust. A holistic approach recognizing health care as a dynamic socio-technical system in which sub-elements interact with each other is necessary to understand trust relationships in human-AI collaboration. For instance, trust in AI systems might be affected by organizational policies, culture, specific tasks assigned to the health care providers, other similar computational tools used by the providers, providers’ interaction with other individuals such as patients and other providers, as well as internal and external environmental factors. This viewpoint is consistent with and complementary to the research roadmaps proposed in the systems engineering literature on AI [45]. Applying human factors methodologies such as the SEIPS model [46] to the health care domain can assist researchers in capturing the entire socio-technical work system. These holistic human factors models provide a useful conceptual framework for researchers to capture contemporary and dynamic issues relevant to trust modeling in healthcare.

Second, recent approvals of AI algorithms reveal the limitations of existing regulatory standards. In early 2018, the FDA authorized the WAVE Clinical Platform, an early-warning system that utilizes vital sign data to identify high-risk patients [35]. FDA standards, designed for traditional rule-based algorithms, do not apply to advanced AI systems whose predictive performance might change when exposed to new data [35]. To measure the impact of AI systems, FDA should benchmark the predictive model and ensure clinically meaningful outcomes. As it does for drug approvals, FDA should rigorously confirm and test surrogate endpoints in potential evaluations of AI systems. Recently, the FDA announced that it is developing a framework for regulating AI systems that self-update on new data and seeking comments on how to regulate self-updating algorithms [47].

AI raises profound concerns regarding medical responsibility. Under current practice, clinicians are only responsible when they deviate from the standard care procedure for a given health condition (irrespective of patient health outcome) [48]. However, clinicians will be held responsible if they follow the AI recommendation when it is different from the standard care process and negatively affects patient health outcomes [48]. What would this mean for an AI system and trust between AI and users? Clinicians using AI systems are expected to use them for decision support, not as a replacement for trained clinical decision-making. In this sense, clinicians are still responsible for any medical errors that may occur as humans remain the final decision-makers. Then, in what capacity can AI assist clinicians and will clinicians be able to use and assess the reliability of an AI system? The influence of these factors on clinicians’ trust in AI applications needs further study.

Considering the limitations of both human cognition and AI approaches, a quantitative measure for the optimal level of trust between clinicians and AI systems to make the most accurate and reliable clinical decisions remains unknown. Linking this optimal level of trust to specific design attributes in an AI system is another unknown. An analysis of that problem should account for the individual human factors specific to the user of the system, including the sizeable aleatory variability associated with it and continuously evolving capabilities of AI methods. The results of such an analysis should inform the regulatory policy decisions.

Finally, trust in AI is expected to have completely different characteristics for patients compared to clinicians. First, patients generally do not have expertise in the medical field, as opposed to clinicians. Further, patients, regardless of whether they are the users of the AI system or not, will be directly impacted by the clinical decisions (or suggestions) that the AI system provides. With the increased patient involvement in a patient-centered health care model, especially educated patients might question clinicians’ decisions and want to be informed whether the decisions are based on AI recommendations or not. If AI systems are an essential part of this shared decision-making process between patients and clinicians, trust relationships between patients and AI systems deserve further in-depth research.

## Abbreviations

AI: artificial intelligence
FDA: Food and Drug Administration
SaMD: Software as a Medical Device
